# The Wnt-β-catenin signaling regulated *MRTF-A* transcription to activate migration-related genes in human breast cancer cells

**DOI:** 10.18632/oncotarget.23961

**Published:** 2018-01-04

**Authors:** Hongpeng He, Fu Du, Yongping He, Zhaoqiang Wei, Chao Meng, Yuexin Xu, Hao Zhou, Nan Wang, Xue-Gang Luo, Wenjian Ma, Tong-Cun Zhang

**Affiliations:** ^1^ Key Laboratory of Industrial Microbiology, Ministry of Education and Tianjin City, College of Biotechnology, Tianjin University of Science and Technology, Tianjin, 300457, P. R. China; ^2^ Department of Pathology, Mentougou Hospital in Beijing, 102300, Beijing, P.R. China; ^3^ College of Life Sciences, Wuhan University of Science and Technology, 430081, Wuhan, P. R. China

**Keywords:** breast cancer, metastasis, MRTF-A, Rho-actin, Wnt-β-catenin

## Abstract

MRTF-A is a transcriptional co-activator being critical for multiple processes including tissue fibrosis and cancer metastasis. The Rho-actin signaling stimulates the nuclear translocation and transcriptional activity of MRTF-A with little effect on the expression of *MRTF-A* gene. High expression of MRTF-A was observed in pancreatic cancer tissues and in TGF-β treated breast cancer cells. However, the mechanism for the upregulation of *MRTF-A* gene remains unclear. In this study, we showed that the transcription of *MRTF-A* was regulated by the Wnt-β-catenin signaling in breast cancer cells. LiCl treatment, Wnt3a treatment or β-catenin overexpression enhanced the transcription of *MRTF-A* gene. In agreement, depletion of β-catenin with siRNA diminished *MRTF-A* transcription. With ChIP assays, β-catenin was identified to interact with the *MRTF-A* promoter whereby it increased histone H4 acetylation and RNA polymerase II association. Further, results of RT-qPCR and Western-blotting supported that the transcriptional co-activator activity of MRTF-A was controlled by both the Rho-actin and the Wnt-β-catenin signaling pathways. MRTF-A was required for cell migration stimulated by the Wnt-β-catenin signaling. Taken together, our results suggest that MRTF-A integrates the Rho-actin and the Wnt-β-catenin signaling to regulate migration-related genes and consequently increases the mobility of breast cancer cells.

## INTRODUCTION

MRTF-A (myocardin-related transcription factor A), also termed MKL1, MAL and BSAC, is a transcriptional co-activator of SRF (serum response factor), which is ubiquitously expressed in a wide range of tissues [1]. The nuclear localization and transcriptional activity of MRTF-A is regulated by the Rho-ROCK-actin signal pathway thereby transducing signals from the cytoskeleton to the nucleus [2–4]. In nuclei, MRTF-A-SRF complex binds to the CArG-box of target promoters to activate transcription [3, 5–8].

In the *Canis familiaris* kidney epithelial (MDCK) cells, MRTF-A was shown to activate the expression of *SLUG* and lead to EMT (epithelial-mesenchymal transition) which is a process highly correlated with cancer metastasis [9]. It was previously reported that MRTFs facilitates breast cancer metastasis by regulating a variety of metastasis-related genes [10]. Recently, MRTF-A was found to be highly expressed in pancreatic cancer tissue [11]. Thus, MRTF-A would be important for the progress of cancer. However, the mechanism by which *MRTF-A* gene is upregulated in cancer cells is largely unknown.

In mouse lung mesenchyme, Wnt2 induced the expression of myocardin and *MRTF-B*, homologs of *MRTF-A*, to promote the development of lung smooth muscle [12]. Although the mechanism was not suggested, this phenomenon inspired us to investigate the role of the Wnt-β-catenin pathway in the expression of *MRTF-A* in breast cancer cells.

Metastasis is a complicated process regulated by multiple signaling pathways. The Wnt-β-catenin pathway which controls the expression of various oncogenes including *c-MYC*, *SNAIL* and *MMPs* (matrix metal proteases) has been extensively studied for its roles in carcinogenesis and metastasis. The Rho-ROCK-actin signaling pathway was also well established for involvement in metastasis. Both the Rho-actin and the Wnt-β-catenin signaling pathways function in metastasis however the relationship between these pathways remains elusive.

In the present study, we showed that the expression of *MRTF-A* was activated by the Wnt-β-catenin pathway. While the Rho-ROCK-actin signaling controlled the transcriptional activity of MRTF-A. Hence, MRTF-A integrated signals from the Rho-ROCK-actin and Wnt-β-catenin pathways to regulate migration-related genes and stimulate breast cancer cell migration.

## RESULTS

### *MRTF-A* gene expression was upregulated by the Wnt-β-catenin signaling

**Figure 1:**
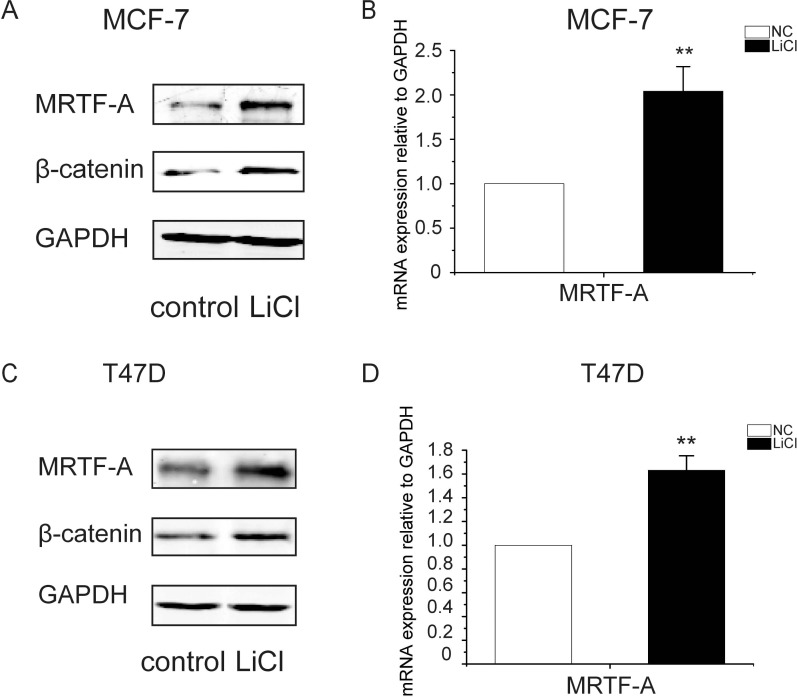
LiCl induced the accumulation of β-catenin protein and the up-regulation of *MRTF-A* transcription in breast cancer cells MCF-7 (**A** and **B**) or T47D (**C** and **D**) cells were treated with 2.5 mM of LiCl for 24 hours before being harvested for Western-blotting or RT-qPCR analysis. (A and C) MRTF-A and β-catenin protein levels increased after LiCl treatment. (B and D) *MRTF-A* mRNA level was upregulated by LiCl. In A and C, figures are representative results of three independent experiments. In B and D, *n* = 3.

To determine the effects of the Wnt-β-catenin signaling on the expression of *MRTF-A*, breast cancer cells MCF-7 were treated with LiCl, an inhibitor of GSK-3β which phosphorylates β-catenin for the ubiquitination-mediated protein degradation. As shown in Figure 1A, the protein level of β-catenin was obviously increased upon LiCl treatment indicating the activation of the Wnt-β-catenin signaling. Under the same condition, the protein level of MRTF-A was augmented as well (Figure 1A) which might be a result of increased protein stability or activated *MRTF-A* gene expression. To measure the expression of *MRTF-A*, RNA was isolated and RT-qPCR was performed. As shown in Figure 1B, the mRNA level of *MRTF-A* was increased by about 2-folds following LiCl treatment, suggesting that the transcription of *MRTF-A* was upregulated by the Wnt-β-catenin signaling. These results were reproduced in another breast cancer cell line T47D (Figure 1C and 1D).

**Figure 2:**
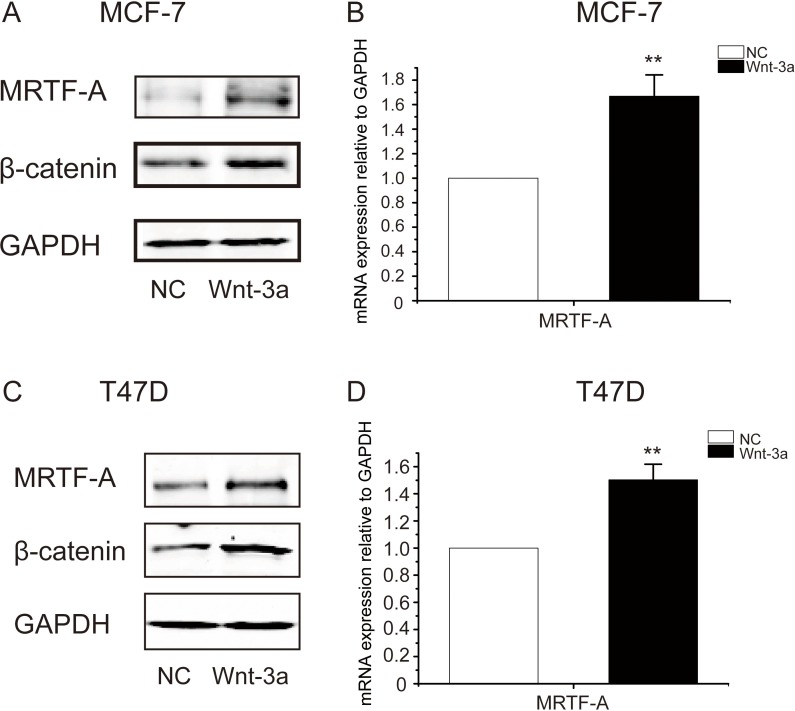
Wnt3a induced the accumulation of β-catenin protein and the up-regulation of *MRTF-A* transcription in breast cancer cells MCF-7 (**A** and **B**) or T47D (**C** and **D**) cells were treated with 100 ng/ml of Wnt3a for 24 hours before being harvested for Western-blotting or RT-qPCR analysis. In A and C, figures are representative results of three independent experiments. In B and D, *n* = 3.

To further examine the effect of the Wnt-β-catenin signaling on *MRTF-A* gene expression, breast cancer cells were treated with Wnt3a, a ligand of Wnt signaling. As shown in Figure 2A and 2C, protein levels of β-catenin and MRTF-A were elevated in Wnt3a-treated cells. The mRNA levels of *MRTF-A* were simoutaneously increased (Figure 2B and 2D). These results support that the Wnt-β-catenin signaling stimulates the expression of *MRTF-A*.

### MRTF-A protein was not stabilized by LiCl

**Figure 3:**
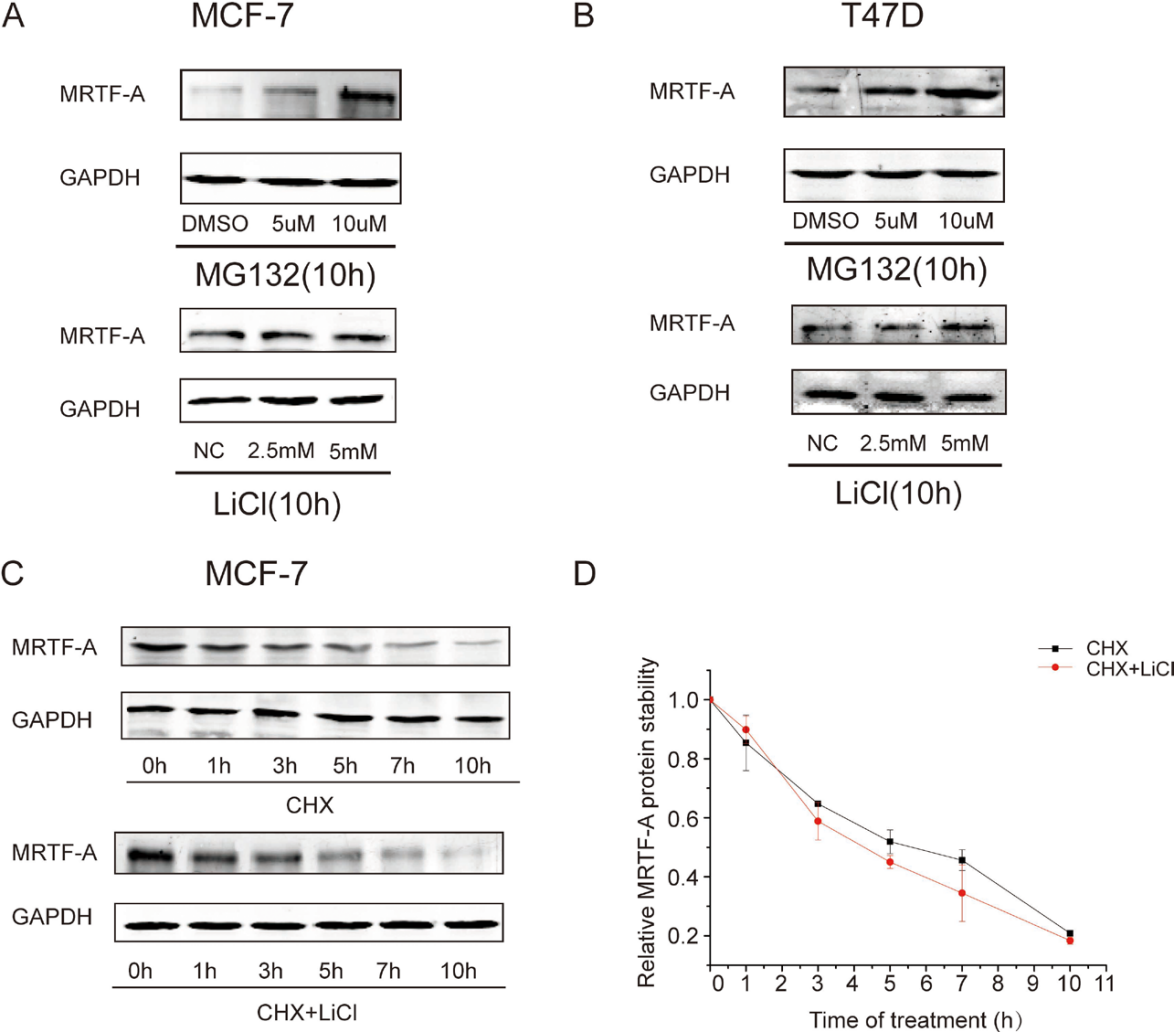
LiCl showed little effect on the stability of MRTF-A protein in breast cancer cells (**A**) MRTF-A protein levels in MG132 or LiCl treated MCF-7 cells. (**B**) MRTF-A protein levels in MG132 or LiCl treated T47D cells. In A and B, cells were treated with MG132 or LiCl at indicated concentrations for 10 h before being harvested for Western-blot analysis. (**C**) MRTF-A protein stability was not changed by LiCl. Protein biosynthesis in MCF-7 cells was blocked with 100 μg/ml of cycloheximide (CHX). MRTF-A protein levels in cells with or without LiCl co-treatment were measured with Western-blot at different time points. (**D**) Quantitative analysis of MRTF-A protein levels presented in C. From A to C, figures are representative results of three independent experiments. In D, *n* = 3.

As a chemical, LiCl may affect cellular processes other than Wnt-β-catenin signaling in breast cancer cells. To test the possibility that LiCl blocks MRTF-A protein degradation, MCF-7 and T47D cells were treated with either LiCl or MG132, an inhibitor of proteasome, for 10 hours. The results of Western-blot showed that MRTF-A protein was significantly accumulated upon MG132 treatment (Figure 3A and 3B, upper panels), indicating that MRTF-A protein is unstable. In contrast, there was little alteration in MRTF-A protein levels following LiCl treatment (Figure 3A and 3B, lower panels), suggesting that LiCl might not block MRTF-A degredation.

Moreover, the half-life of MRTF-A protein was measured. Cycloheximide was added into culture media to inhibit protein synthesis. Once the biosynthesis of MRTF-A protein was suppressed by cycloheximide, cellular MRTF-A protein level decreased gradually (Figure 3C, upper panel). Co-treatment with LiCl and cycloheximide showed a similar pattern of MRTF-A protein level (Figure 3C, lower panel). Quantitative analysis of MRTF-A protein half-life showed no significant difference between LiCl treated cells and the controls (Figure 3D), demonstrating that LiCl did not increase MRTF-A protein stability.

### LiCl elevated the expression of MRTF-A target genes

**Figure 4:**
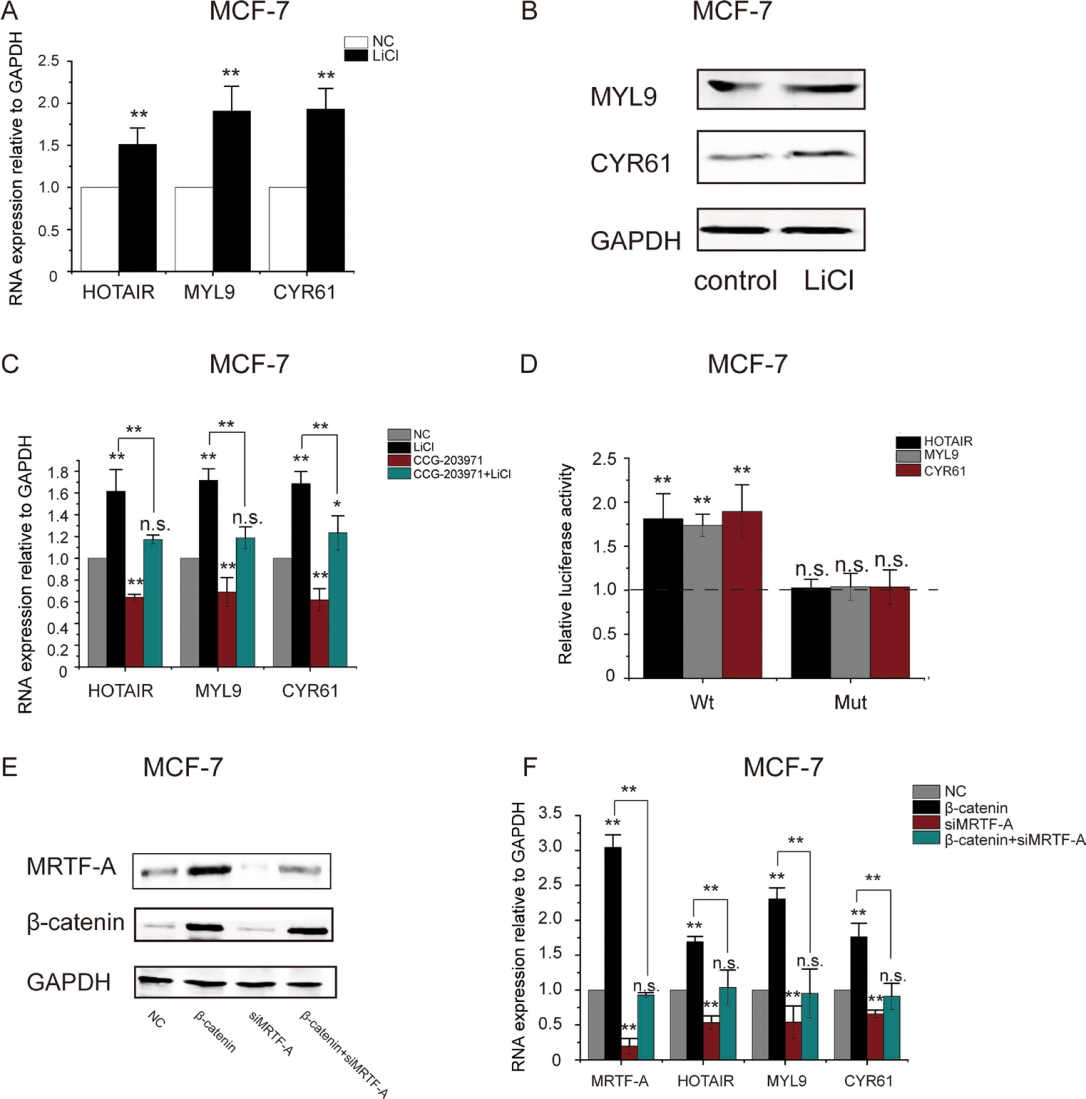
LiCl induced the activation of MRTF-A target genes in an MRTF-A-dependent manner In (**A**) and (**B**), MCF-7 cells were treated with LiCl as described in Figure 1. (A) The mRNA of MRTF-A target genes *HOTAIR*, *MYL9* and *CYR61* was significantly upregulated by LiCl. (B) MYL9 and CYR61 protein levels were upregulated by LiCl. Western-blot was performed with GAPDH as the loading control. (**C**) MRTF-A inhibitor CCG-203971 suppressed the LiCl-induced upregulation of *HOTAIR*, *MYL9* and *CYR61* in MCF-7 cells. Cells were treated with 10 μM of CCG-203971 in combination with 2.5mM of LiCl or not as indicated. (**D**) LiCl increased *HOTAIR*, *MYL9* and *CYR61* promoter activities in a CArG-dependent manner. Activities of the wild-type or CArG-mutated promoters without LiCl-treatment were set as one. Promoter activities in LiCl-treated cells were normalized to the corresponding untreated controls and were plotted in the bar graph. (**E**) Depletion of MRTF-A protein with *MRTF-A*-specific siRNA in MCF-7 cells with or without LiCl co-treatment. (**F**) RNA levels of MRTF-A target genes in *MRTF-A* knockdown cells. In A, C and F, *n* = 3. In D, *n* = 6. In B and E, figures are representative results of three independent experiments.

MRTF-A is a co-activator directing the transcription of both protein coding and non-coding genes. To examine the transcriptional activity of MRTF-A, expression of MRTF-A target genes *MYL9*, *CYR61* and lncRNA *HOTAIR* was determined. As shown in Figure 4A, the RNA levels of both protein-coding genes *MYL9* and *CYR61* and the non-coding gene *HOTAIR* were significantly increased in LiCl-treated MCF-7 cells. In agreement, the protein levels of MYL9 and CYR61 were higher following LiCl-treatment (Figure 4B), suggesting that the expression of MRTF-A target genes was activated by LiCl.

To determine the role of MRTF-A in the LiCl-induced upregulation of *MYL9*, *CYR61* and *HOTAIR* genes, MCF-7 cells were treated with CCG-203971, an inhibitor of MRTF-A activity [13]. The results of RT-qPCR showed that CCG-203971 counteracted the stimulating effect of LiCl on the transcription of *MYL9*, *CYR61* and *HOTAIR* genes (Figure 4C), suggesting that MRTF-A was required for the activation of *MYL9*, *CYR61* and *HOTAIR* genes induced by LiCl.

To further demonstrate the participation of MRTF-A in LiCl-stimulated activation of *MYL9*, *CYR61* and *HOTAIR genes*, activities of wild type or CArG-mutated *MYL9*, *CYR61* and *HOTAIR* promoters were analyzed with luciferase assays. As shown in Figure 4D, activities of the wild type promoters were significantly augmented after LiCl-treatment however the MRTF-A-binding site CArG-mutated promoters showed no response to LiCl, indicating that MRTF-A was important for the LiCl-induced gene activation.

Depletion of MRTF-A was carried out with specific siMRTF-A. As shown in Figure 4E, without LiCl, MRTF-A protein was efficiently depleted by siMRTF-A (Figure 4E, compare lane 3 with lane 1). With LiCl, the expression of MRTF-A was remarkably enhanced so that MRTF-A protein level was largely reduced but still detectable after siMRTF-A interference (Figure 4E, compare lane 4 with lane 2). Following MRTF-A knockdown, the elevated RNA levels of *HOTAIR*, *MYL9* and *CYR61* in LiCl-treated cells were dramatically decreased (Figure 4F), confirming that MRTF-A mediated the LiCl-induced upregulation of *HOTAIR*, *MYL9* and *CYR61.*

Taken together, these results suggest that the Wnt-β-catenin signaling stimulates the expression of MRTF-A which activates the transcription of metastasis-related genes.

### β-catenin directly activated the transcription of *MRTF-A* gene

**Figure 5:**
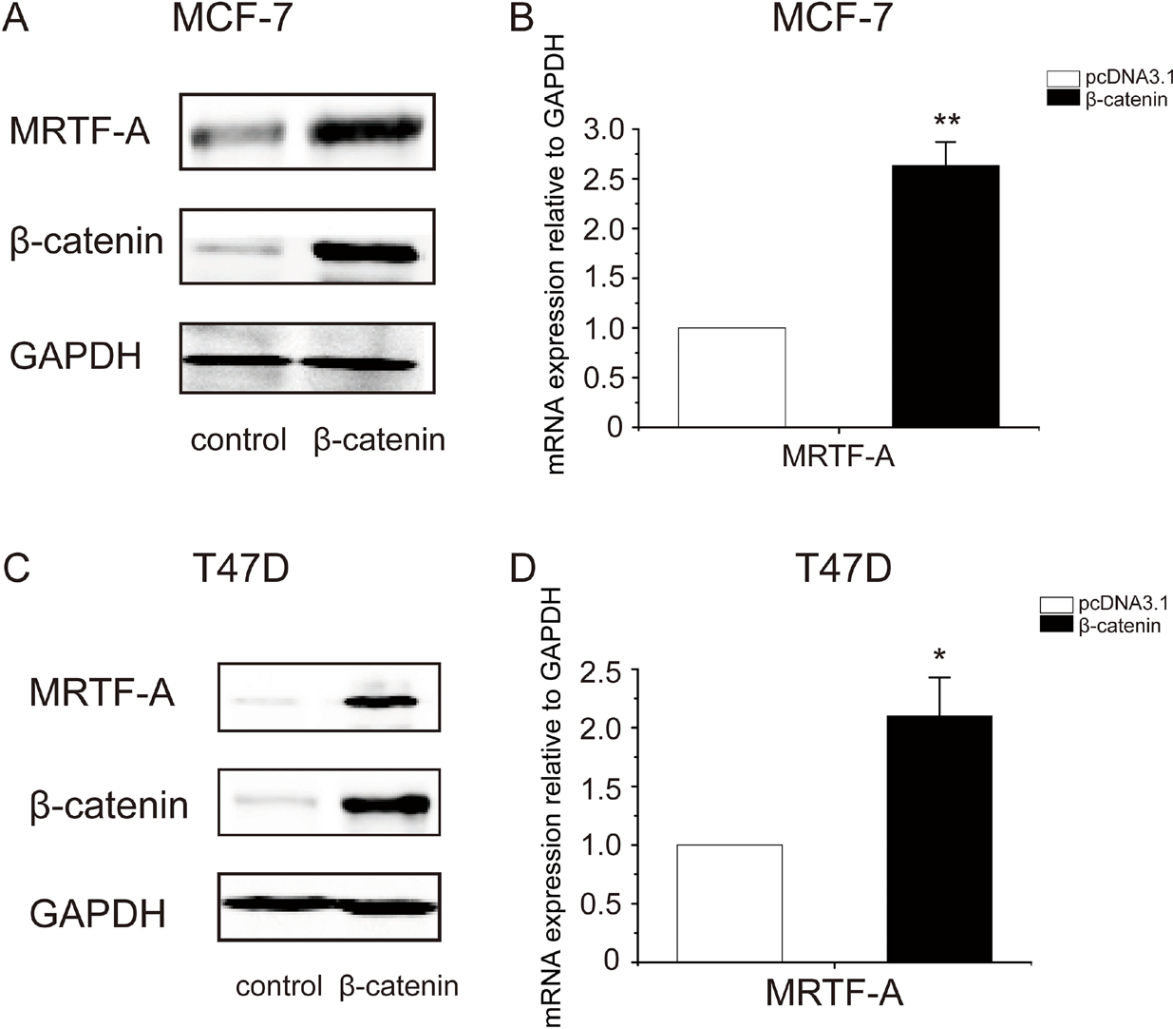
Overexpression of β-catenin elevated the transcription of MRTF-A gene (**A**) The successful overexpression of β-catenin increased MRTF-A protein level in MCF-7 cells (**B**) Overexpression β-catenin augmented the mRNA levels of *MRTF-A* in MCF-7 cells. pcDNA3.1 served as empty vector control. (**C**) Overexpression of β-catenin increased the protein level of MRTF-A in T47D cells (**D**) β-catenin augmented *MRTF-A* transcription in T47D cells. In A and C, figures are representative results of three independent experiments. In B and D, *n* = 3.

To explore the mechanism by which LiCl enhanced *MRTF-A* expression, β-catenin was overexpressed in breast cancer cells. The efficacy of β-catenin overexpression was visualized with Western-blot (Figure 5A, middle panel). Using the same samples, MRTF-A protein was determined. The results showed that MRTF-A protein level was increased upon β-catenin overexpression (Figure 5A, top panel), suggesting that β-catenin upregulates the expression of *MRTF-A* gene.

Further, the mRNA level of *MRTF-A* was measured with realtime qPCR. The results showed that *MRTF-A* mRNA level was elevated by β-catenin (Figure 5B), indicating that β-catenin activates the transcription of *MRTF-A* gene. Similar results were observed in T47D breast cancer cells (Figure 5C and 5D), supporting a positive role of β-catenin in the transcription of *MRTF-A*.

**Figure 6:**
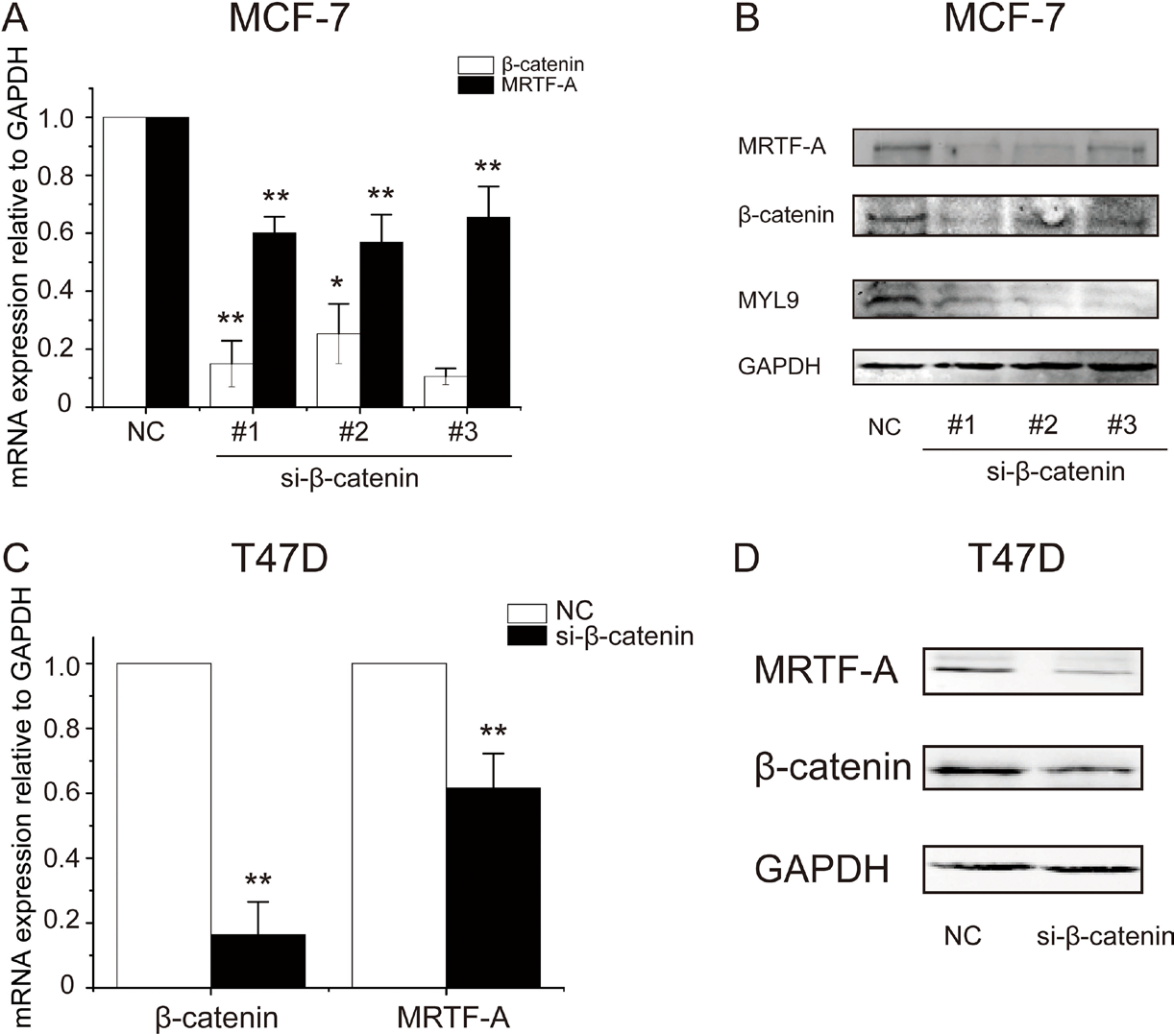
β-catenin depletion diminished the transcription of MRTF-A gene (**A**) Knockdown efficiency of three different si-β-catenin sequences and the simultaneously downregulated *MRTF-A* mRNA levels in MCF-7 cells. (**B**) Examination of the efficacy of β-catenin knockdown and the expression of MRTF-A and MYL9 at protein level in MCF-7 cells. (**C**) β-catenin depletion downregulated the expression of *MRTF-A* at mRNA level in T47D cells. (**D**) Western-blot analysis of the efficacy of β-catenin depletion and the expression of MRTF-A at protein level in T47D cells. In C and D, depletion of β-catenin was carried out with si-β-catenin #1. In A and C, *n* = 3. In B and D, figures are representative results of three independent experiments.

To confirm the influence of β-catenin on *MRTF-A* expression, β-catenin was depleted with specific siRNAs. The efficacy of depletion was approximate 80% as evaluated with RT-qPCR, meanwhile, *MRTF-A* mRNA levels were reduced by about 40% (Figure 6A). In line with the mRNA levels, MRTF-A protein levels were obviously decreased following β-catenin knocked-down (Figure 6B). Under the same condition, the protein expression of MRTF-A target gene *MYL9* was nearly abolished (Figure 6B) suggesting deficiency in MRTF-A activity. The inhibitory effect of β-catenin depletion on *MRTF-A* expression was reproduced in T47D cells (Figure 6C and 6D), supporting that β-catenin was required for the transcription of *MRTF-A* gene.

### β-catenin physically interacted with *MRTF-A* gene promoter

**Figure 7:**
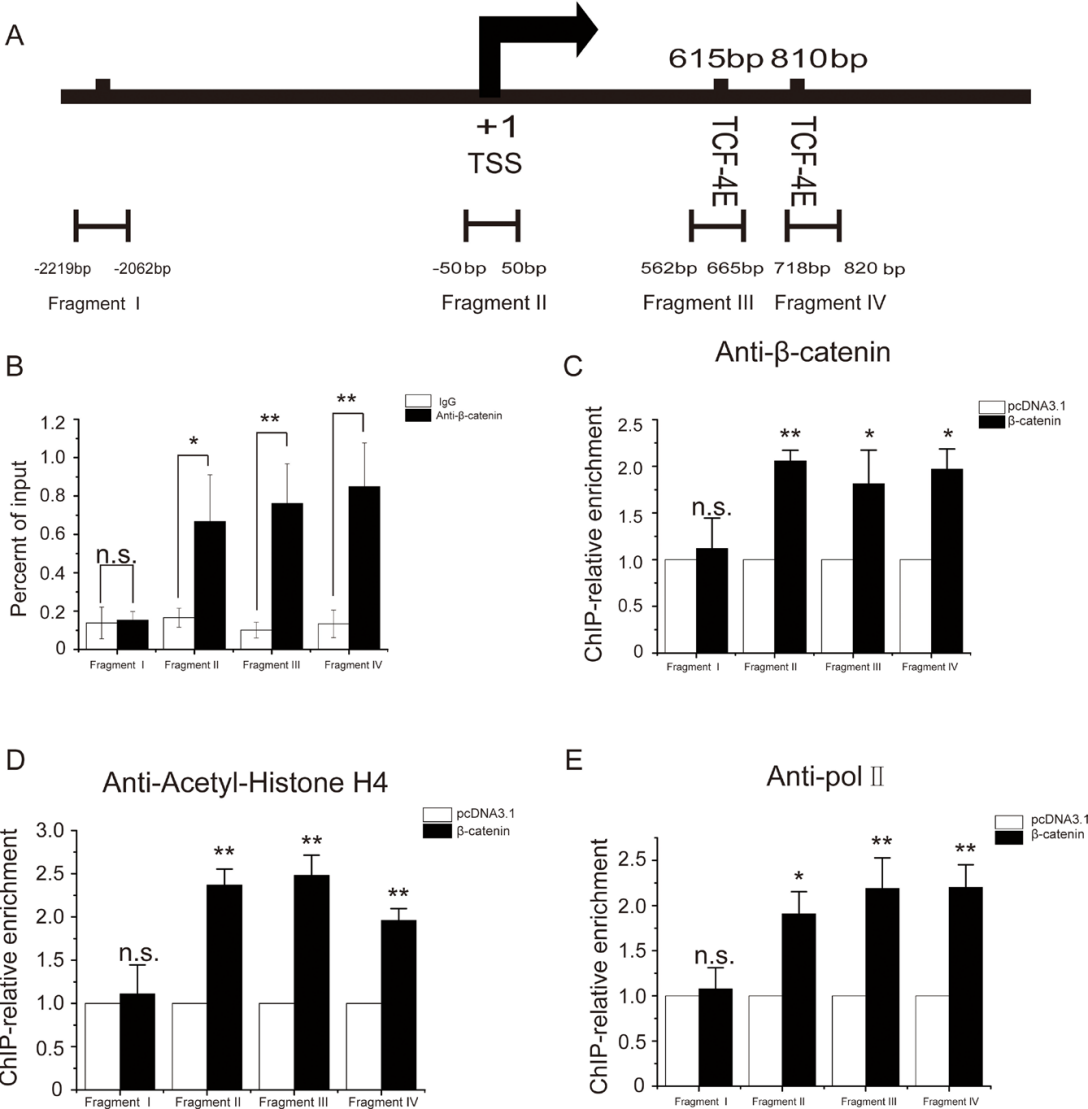
β-catenin physically associated with the promoter of MRTF-A to facilitate histone acetylation and RNA Polymerase II recruitment (**A**) Schematic of the *MRTF-A*-promoter with the positions of four ChIP-PCR fragments depicted. Fragment I is about 3 kb upstream of the TCF-4E binding sites. Fragment II contains the transcription start site (TSS) and is in the core promoter of *MRTF-A* gene. Fragments III and IV cover the TCF-4E sequences. (**B**) β-catenin associated with the promoter of *MRTF-A*. ChIP was performed with β-catenin antibodies and ChIP products were quantatively analized of with qPCR. (**C**) β-catenin association on *MRTF-A* promoter increased following β-catenin overexperssion (**D**) β-catenin overexpression enhanced the acetylation of histone H4 at the *MRTF-A* promoter. (**E**) Overexpression of β-catenin enhanced the recruitment of RNA polymerase II to the *MRTF-A* promoter. From B to E, *n* = 4.

β-catenin is a transcriptional co-activator interacting with target promoters via the bridging of transcription activators LEF/TCF. There are two T cell factor (TCF-4E) binding sequences near the transcription start site (TSS) of *MRTF-A* gene (Figure 7A). To test whether β-catenin directly regulates *MRTF-A* gene, ChIP assays were performed with antibodies against β-catenin. Figure 7A shows the positions of PCR fragments amplified in the ChIP assays. Fragment I is a negative control which is at 3 kb upstream of the TCF-4E binding sequences. Fragment II is at the core promoter covering the TSS. Fragments III and IV correspond to the two TCF-4E binding sites, respectively. Figure 7B shows the physical association of endogenous β-catenin with the *MRTF-A* gene promoter, indicating a direct role of β-catenin in *MRTF-A* gene regulation.

In order to explore the mechanism by which β-catenin promotes *MRTF-A* expression, β-catenin was overexpressed and ChIP assays were performed with antibodies as indicated. Following β-catenin overexpression, the association of β-catenin became more enriched (Figure 7C). Under the same condition, the acetylation of histone H4 in the *MRTF-A* gene promoter was increased (Figure 7D) suggesting a more permissive chromatin structure. Meanwhile, there was more RNA polymerase II associated with the *MRTF-A* gene promoter (Figure 7E) which was in line with the upregulation of *MRTF-A* gene by β-catenin overexpression.

These data, taken together, indicate that β-catenin favors the acetylation of histones and facilitates the recruitment of RNA polymerase II to the *MRTF-A* promoter to upregulate *MRTF-A* transcription.

### Rho-actin and Wnt-β-catenin signaling pathways corporately activated MRTF-A target genes

**Figure 8:**
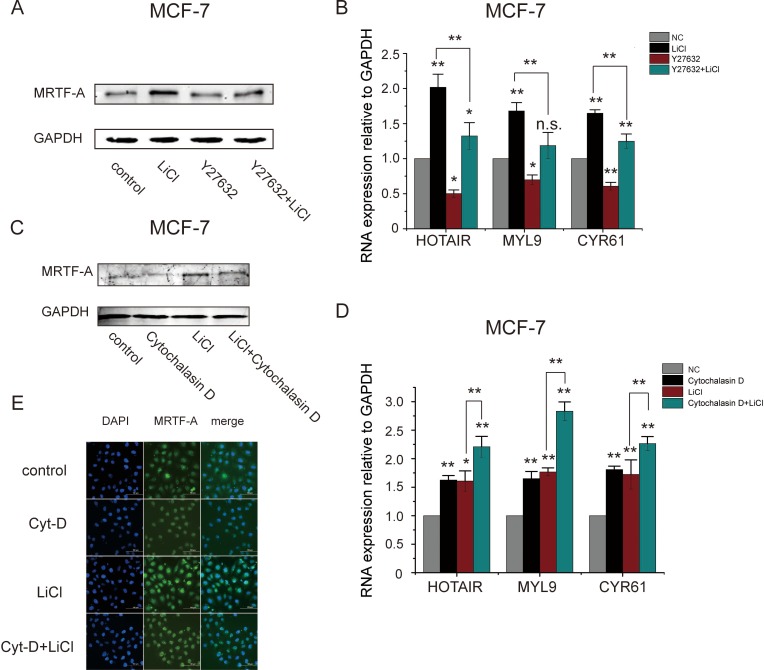
Rho-ROCK-actin signaling was crucial for the LiCl-induced MRTF-A transactivity (**A**) The expression of MRTF-A after treatment with 25 μM of Y27632, 2.5 mM of LiCl or co-treatment with Y27632 and LiCl for 24 hours in MCF-7 cells. (**B**) The expression of MRTF-A target genes in MCF-7 cells following treatment with Y27632 or LiCl as indicated. (**C**) The expression of MRTF-A in MCF-7 cells after treatment with 2.5 mM of LiCl for 24 hours or co-treatment with 5 μM of cytochalasin D for 6 hours. (**D**) The expression of MRTF-A target genes in MCF-7 cells following treatment with cytochalasin D or LiCl as indicated. (**E**) Cellular distribution of MRTF-A protein in MCF-7 cells treated with cytochalasin D and/or LiCl. Figures A C and E are representative results of three independent experiments. In B and D, *n* = 3.

It was previously established that the Rho-ROCK-actin signaling pathway is important for the transcriptional activity of MRTF-A. In the present study, *MRTF-A* expression was identified to be stimulated by the Wnt-β-catenin signaling pathway. Thus, MRTF-A might integrate signals from both pathways to regulate downstream metastasis-related genes. To test this idea, MCF-7 cells were co-treated with LiCl and Y27632, an inhibitor of ROCK. As shown in Figure 8A, MRTF-A protein level was increased after LiCl treatment in spite of co-treatment with Y27632 or not. The transcription of MRTF-A target genes *HOTAIR*, *MYL9* and *CYR61* was induced by LiCl but inhibited by Y27632. Co-treatment of Y27632 with LiCl reversed the LiCl-induced upregulation of MRTF-A target genes (Figure 8B), indicating that the upregulation of MRTF-A expression dictated by the Wnt signaling was not sufficient for the fully activation of downstream target genes, signal from the Rho-ROCK-actin signaling pathway was also required for MRTF-A transcriptional activity.

Cytochalasin D inhibits G-actin binding to MRTF-A protein thus facilitates MRTF-A nuclear translocation. As shown in 8C, single treatment of LiCl but not cytochalasin D elevated MRTF-A protein level. Co-treatment of MCF-7 cells with cytochalasin D and LiCl did not further increase the protein level of MRTF-A (Figure 8C, compare lane 4 with lane 3). As shown in Figure 8D, single treatment of LiCl or cytochalasin D increased the transcription of MRTF-A target genes which was further enhanced by the co-treatment (Figure 8D), suggesting that the expression of MRTF-A target genes was coordinately regulated by the Rho-ROCK-actin and the Wnt-β-catenin signaling pathways.

Figure 8E shows the immunoflorence detection of MRTF-A protein. Without LiCl or cytochalasin D, MRTF-A was distributed in nuclei and perinuclear area in breast cancer MCF-7 cells (the first row). With cytochalasin D, cytoplasmic MRTF-A was translocated into nulei (the second row). With LiCl, the florescent signals for MRTF-A protein were strengthened however the cellular distribution was not changed (the third row). In cytochalasin D and LiCl co-treated cells, MRTF-A was localized in nuclei (the bottom row), indicating that the Rho-ROCK-actin signaling controlled the cellular localization of MRTF-A protein and the Wnt-β-catenin signaling regulated MRTF-A expression.

Taken together, the above results suggest that the Rho-ROCK-actin and the Wnt-β-catenin signaling pathways corporately control the MRTF-A-activitied gene expression.

### MRTF-A was important for the cell migration induced by the Wnt-β-catenin signaling pathway

**Figure 9:**
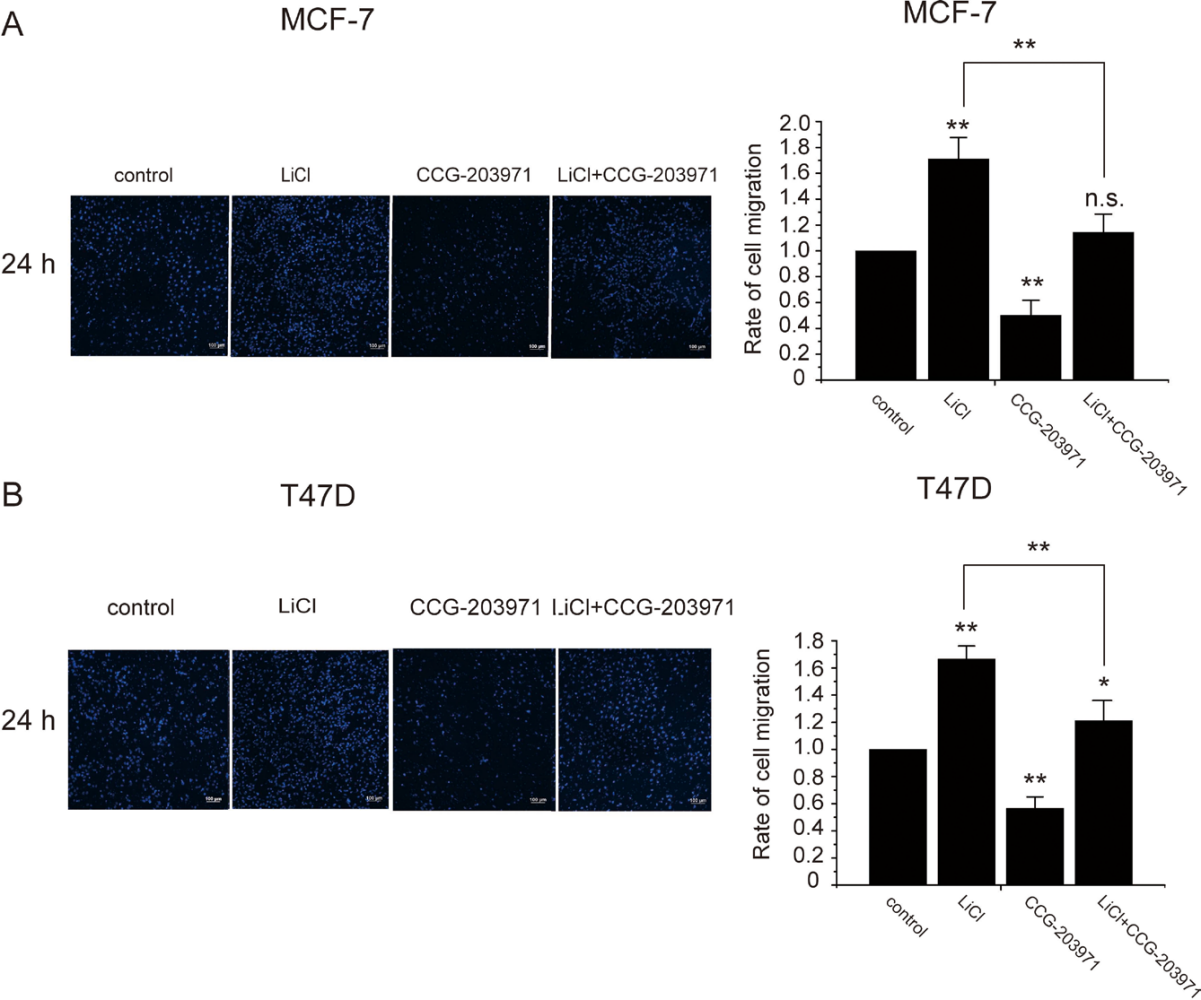
MRTF-A transactivity was required for the LiCl-induced migration of breast cancer cells Transwell migration assays after incubation with 10 μM of CCG-203971, 2.5 mM of LiCl or both CCG-203971 and LiCl as indicated. The left panels show the DAPI staining of the membranes. The right panels show the quantitatively analysis of cell migration rates. (**A**) Transwell migration assays of MCF-7 cells. (**B**) Transwell experiments with T47D cells. Migration rates were calculated with six independent experiments, *n* = 6.

The Wnt-β-catenin signaling pathway was known to induce cancer metastasis but its role in cancer cell migration was controversial [14, 15]. To examine the influence of the Wnt-β-catenin signaling on MCF-7 cell migration and the involvement of MRTF-A, transwell cell migration assays were performed. As shown in Figure 9A, LiCl treatment promoted the migration of breast cancer cells across the membrane whereas CCG-203971, an inhibitor of MRTF-A, suppressed the trans-membrane migration. Co-treatment with LiCl and CCG-203971 partially reversed the migration induced by LiCl suggesting that MRTF-A activity is required for the LiCl-induced cell migration. In T47D breast cancer cells, similar phenomena were observed (Figure 9B), supporting the involvement of MRTF-A in cell migration induced by the Wnt signaling.

**Figure 10:**
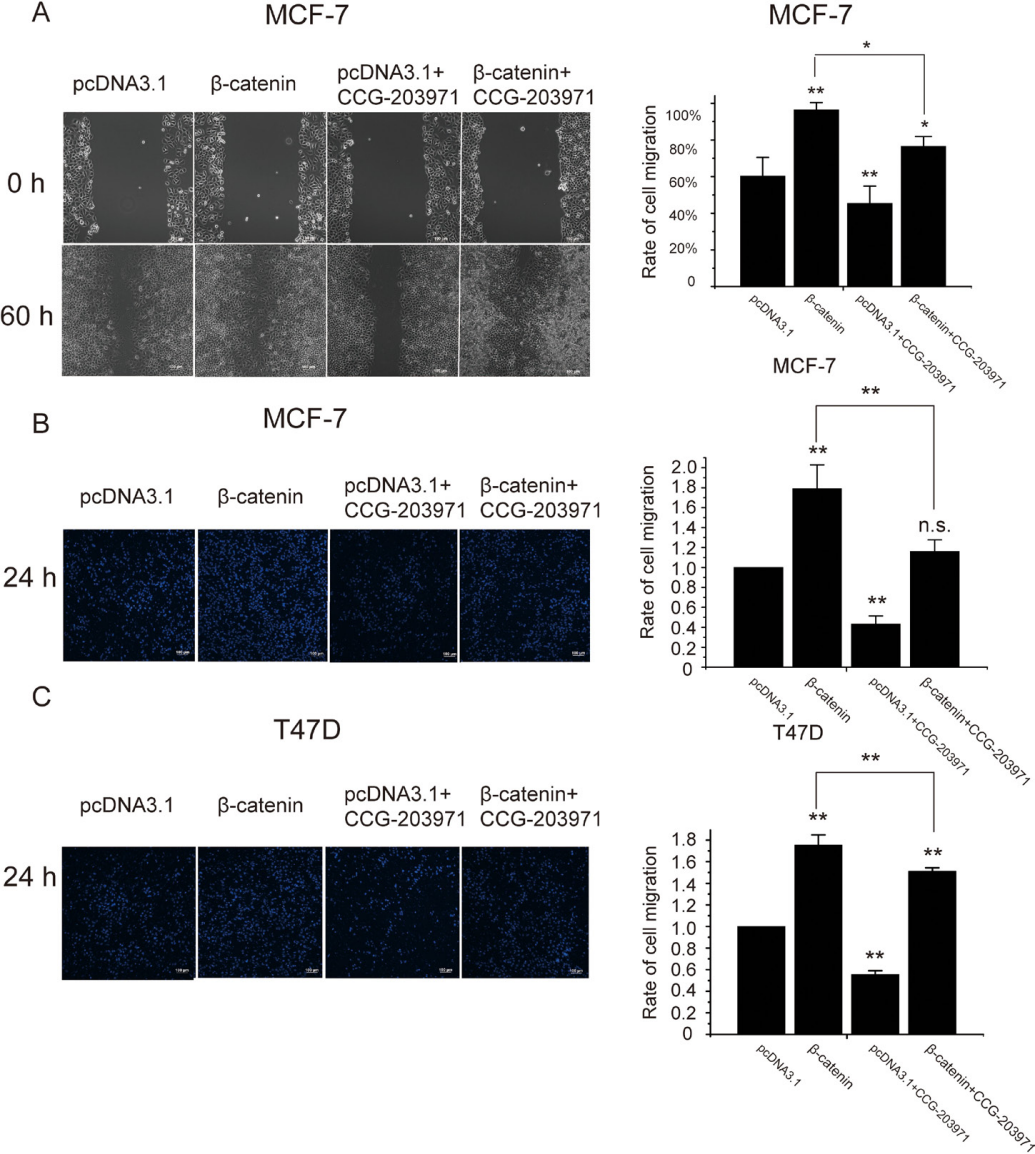
MRTF-A transactivity was required for the β-catenin-stimulated migration of breast cancer cells Cells were treated with 10 μM of CCG-203971 in combination with or without the transfection of β-catenin-encoding plasmids as indicated. The right panels show the quantitatively analysis of cell migration rates. (**A**) Results of Scratch wound healing experiments show the parallel migration of MCF-7 cells. (**B**) Results of Transwell experiments show the transmembrane migration of MCF-7 cells. (**C**) Results of Transwell experiments show the transmembrane migration of T47D cells. Migration rates were calculated with six independent experiments, *n* = 6.

To further confirm the role of Wnt signaling in breast cancer cell migration, MCF-7 and T47D cells were transfected with β-catenin encoding plasmids. In scratch wound-healing assays, cell mobility was evaluated with the rate of wound-healing. As shown in Figure 10A, β-catenin overexpression enhanced wound-healing which was significantly inhibited by CCG-203971, supporting the involvement of MRTF-A in the β-catenin mediated cell migration. The results of transwell assays showed that overexpression of β-catenin stimulated the trans-membrane cell migration and co-treatment with CCG-203971 attenuated cell mobility (Figure 10B and 10C), demonstrating that MRTF-A was important for the β-catenin induced cell migration.

Overall, the results of transwell migration and scratch wound healing assays demonstrated the requirement of MRTF-A for cell migration stimulated by the Wnt-β-catenin signaling pathway.

## DISCUSSION

MRTF-A was previously identified to promote EMT, a process leading to fibrosis, during physiological wound healing and in some malignant and benign diseases. Based on this, a serial of MRTF-A inhibitors have been developed and investigated to prevent scar formation or tissue fibrosis in a variety of tissues such as skin, lung, eye and colon [16–19]. MRTF-A inhibitor was recently reported to block lung metastases of Rho C-overexpressing melanoma [20], suggesting MRTF-A to be a potential target of cancer therapy.

In LLC-PK1, a porcine proximal tubular epithelial cell line, β-catenin was shown competed with Smad3 to interact with MRTF-A/SRF complex and thus stabilized MRTF-A from Smad3-mediated degradation [21]. In MS-1 endothelial cells, Smad was suggested to be required for the TGF-β induced activation of *MRTF-A* gene [22]. It seems that the roles of β-catenin and Smad3 in MRTF-A protein degradation or *MRTF-A* gene regulation are cell type-dependent. In the present study, LiCl induced accumulation of β-catenin and increased *MRTF-A* gene expression in human breast cancer cells (Figure 1) without altering the stability of MRTF-A protein (Figure 3). β-catenin overexpression or depletion enhanced or diminished the transcription of *MRTF-A* gene, respectively (Figures 5 and 6). In addition, β-catenin protein associated with the promoter of *MRTF-A* gene and augmented the recruitment of RNA polymerase II (Figure 7). Thus, we propose that β-catenin functions as a co-activator of *MRTF-A* gene in human breast cancer cells.

Our previous study proved that the Rho signaling stimulated the transactivity of MRTF-A without affecting its expression [23]. In the present study, we unraveled a novel mechanism of *MRTF-A* gene regulation by the Wnt-β-catenin signaling pathway in breast cancer cells. Our data showed that *MRTF-A* gene was activated by the Wnt-β-catenin signaling, in particular, by β-catenin which physically associated with *MRTF-A* gene promoter. With the expression being regulated by the Wnt-β-catenin signaling and the nuclear localization being regulated by the Rho-ROCK-actin signaling, MRTF-A may integrate signals from the above two signaling pathways to promote the expression of migration-related genes in breast cancer cells.

Our findings herein establish a link between the well defined Rho-actin and Wnt-β-catenin signaling pathways, which would contribute to a better understanding to the mechanism of breast cancer metastasis. As reviewed, multiple signaling pathways including TGF-β-Smad, cytokine-JAK-STAT, growth factor – RTK-Ras are involved in EMT and tumor metastasis [24]. Whether MRTF-A also integrates signals from these signaling pathways would be worth of further study to learn the importance of MRTF-A in cancer metastasis.

## MATERIALS AND METHODS

### Cell culture and transfection

Human breast cancer MCF7 and T47D cells were cultured in Dulbecco’s modified Eagle’s medium with L-Glutamine (Gibico) supplemented with 10% fetal bovine serum (Kangyuan, Tianjin, China) at 37°C in a humidified incubator with 5% CO_2_. The wild type and mutated pGL3-HOTAIR, pGL3-MYL9 and pGL3-CYR61 luciferase reporter plasmids were previously described [23, 25]. A human β-catenin encoding plasmid was constructed with the pCMV-Tag2B as backbone in this study. Plasmids were transiently transfected into cells with TurboFect (Thermo, USA) or FuGENE HD Transfection Reagent (Promega) following the manufacturer’s instructions. The β-catenin interfering siRNA duplexes, designed and synthesized by RiboBio, Guangzhou, China, were introduced into cells with RiboFect transfection agent (RiboBio, Guangzhou, China). Wnt3a was purchased from R&D Systems. Cycloheximide was from MedChemExpress. Cytochalasin D, MG132 and the ROCK inhibitor Y27632 were purchased from Sigma. MRTF-A inhibitor CCG-203971 was purchased from *ApexBio* Technology, USA.

### Extraction of total RNA and RT-qPCR

Total RNA was extracted with Trizol (Invitrogen). cDNA was synthesized with M-MLV reverse transcriptase (Promega) and quantified by realtime qPCR using Biosystems StepOne™ Real-Time PCR system and Fast SYBR Green Master Mix (Promega). PCR primers were designed with NCBI online software Primer-BLAST and synthesized by Invitrogen. The sequences of primers used in this study are listed in Table 1.

**Table 1:** The sequences of primers used in realtime-qPCR in this study

Genes	Primer sequence
GAPDH	F:CGAGATCCCTCCAAAATCAA
	R:TTCACACCCATGACGAACAT
MRTF-A	F:ACCGTGACCAATAAGAATGC
	R:CCGCTCTGAATGAGAATGTC
HOTAIR	F:TAGGCAAATGTCAGAGGGTT
	R:ACACAAGTAGCAGGGAAAGG
β-catenin	F:ACCAGTGGATTCTGTGTTGTT
	R:ATTTGAAGGCAGTCTGTCGTA
MYL9	F:GAGCCCAAGCGCCTTCT
	R:GTCAATGAAGCCATCACGGT
Cyr61	F:AAGGATAGTATCAAGGACCCC
	R:ATCCATTCCAAAAACAGGGAG
ChIP-fragment I	F:CCAACCTGGTCTCTAACT
	R:CACACCCACTATCTCAC
ChIP-fragment II	F:CCTCCCCCAGAGACGAAT
	R:ACTGCCGACAGGAAACCA
ChIP-fragment III	F:GAATTAGATGGTTACTGGT
	R:GTGCCACAGACAATACA
ChIP-fragment IV	F:TGGTTCTGTGGAAATGCCC
	R:CAAAAATCCTCCCCTGTGT

### Western-blotting analysis

Proteins in whole cell lysates were separated with SDS-PAGE and then transferred to nitrocellulose membrane (Millipore). Primary antibodies including β-catenin (Beyotime, China, AF0069), MRTF-A (proteintech, 21166-1-AP), Cyr61(Santa Cruz, sc-374129), MYL9 (Santa Cruz, sc-28329) and GAPDH (Santa Cruz). Secondary antibodies were IRDye-conjugated donkey anti-mouse or anti-rabbit IgG (Licor Biosciences) and membranes were visualized with Odyssey Infrared Imaging System (Gene Company Limited).

### Luciferase reporter assay

The wild type or CArG-mutated luciferase reporter plasmids were transfected into MCF-7 cells 24 h before LiCl treatmemt for another 24 h. Cells were lyzed and luciferase activities were measured with Luciferase Assay System (Promega) in a SynergyTM 4 luminometer (Bioteck).

### Chromatin immunoprecipitation (ChIP)

ChIP assays were carried out following the standard protocol. Briefly, MCF7 cells were cross-linked with 1% of formaldehyde (Sigma) for 15 min at room temperature with gentle shaking. Cells were harvested with cell scraper and chromatin was fragmentized to average 300 bp in length with sonicator. Diluted whole cell sonicates were incubated with anti-β-catenin (Beyotime, China, AF0069), anti-RNAP II (Covance, MMS-126R-200) or anti-acH4 (Millipore, 06-866) antibodies with normal serum IgG as controls. After protein A beads (Millpore, 17-295) binding, washing and eluting, ChIP products were purified with phenol/chloroform and measured by real-time qPCR.

### Scratch wound healing assay

Scratch wound healing assays were performed as described previously [25]. When cells were grew to confluence, cell monolayers were scratched by a sterile 10 μl pipette tip and then cultured with medium containing LiCl or CCG-203971 as indicated in figure legends. At 12 h time intervals, the healing of the wound was investigated and photographed with a microscope.

### Transwell cell migration assay

MCF-7 or T47D cells were diluted with DMEM-F12 culture media containing 0.5% FBS to 5 × 10^8^ /L before being seeded into the upper chamber of Transwell (Corning). 0.6 ml of DMEM-F12 medium containing 10% FBS was added into the lower chamber before being cultured in an incubator. 24 h after the incubation, cells attached to the upper surface of the membrane were wiped out with cotton swabs. The membrane was fixed with 4% of paraformaldehyde and stained with DAPI to visualize the cells penetrating membrane. The number of cells was analyzed with Image J.

### Immunofluorescence staining

Cells were fixed with 4% of paraformaldehyde for 30 minutes and then permeabilized with 0.3% Triton X-100 for 15 minutes. The cells were sequentially incubated with MRTF-A antibodies and FITC-labeled secondary antibodies and washed three times with PBS and counterstained with DAPI (ThermoFisher, USA). The cells were analyzed using a confocal laser scanning microscope (Leica TCS-NT, Germany). Each sample has at least three separate random fields captured from three independent experiments.

### Statistical analysis

All statistical analyses were performed using SPSS 17.0 (SPSS, Chicago, USA) using either one-sample *t*-test or one-way ANOVA analysis. All data were presented as mean ± S.E.M. *p* value of less than 0.05 was indicated with ^*^, less than 0.01 was indicated with ^**^.
